# The burden of rheumatic heart disease and issues affecting the provision of care in Malawi: A scoping review

**DOI:** 10.1371/journal.pntd.0013400

**Published:** 2025-08-19

**Authors:** Eva Blennerhassett, Oisin Brady Bates, Maire O’Connor, Hastings Gondwe, Lillian Msimuko, Mark Ledwidge, Balwani Mbakaya, Joseph Gallagher

**Affiliations:** 1 UCD School of Medicine, University College Dublin, Dublin, Ireland; 2 Department of General Practice, UCD School of Medicine, University College Dublin, Dublin, Ireland; 3 Non-Communicable Disease Clinic, St John’s Hospital, Mzuzu, Malawi; 4 Department of Public Health, University of Livingstonia, Livingstonia, Malawi; University of Connecticut College of Agriculture Health and Natural Resources, UNITED STATES OF AMERICA

## Abstract

**Background:**

Rheumatic heart disease (RHD) is an autoimmune sequela of group A streptococcal (GAS) pharyngitis. Acute rheumatic fever (ARF), a complication arising 2–3 weeks after GAS infection, can cause damage to the valves of the heart and, ultimately, RHD. This trajectory towards severe disease is now rare in high-income countries. In Malawi, as in many low-income countries, RHD continues to pose a significant challenge. Limited access to healthcare and poor education likely contribute to the disease burden. This scoping review aims to determine the present burden of GAS, ARF and RHD in Malawi, the issues affecting the provision of care and the solutions that have been proposed.

**Method:**

A search was conducted of ‘PubMed’, ‘EMBASE’, ‘Cochrane Library’ and ‘Clinicaltrials.gov’ to identify research published between 1995 and 2024 according to the Arksey and O’Malley framework. The PRISMA extension for Scoping Review (PRISMA-ScR) Checklist guided the search (Supplementary File 3).

**Results:**

Data were extracted from thirty articles. RHD prevalence among Malawian children ranges between 3.4% and 5.3%, with high rates of late presentation (62%-82.5% presenting with severe disease). Inadequate health system infrastructure, limited RHD-specific education for healthcare providers, and inconsistent availability of benzathine penicillin G for secondary prophylaxis were identified as key challenges. Rural areas, comprising 84% of Malawi’s population, are particularly underserved. Task-shifting to non-physician healthcare workers in primary care has shown promise. Much less research was available on ARF and GAS infections.

**Conclusion:**

The significant morbidity and mortality associated with RHD is a major concern in the communities and healthcare systems of Malawi. Primary care resourcing and improved education are areas requiring attention. To address the high burden of disease in the country, ongoing research is largely focused on establishing a sufficiently large and appropriately trained workforce to diagnose and monitor RHD using the resources available within the constraints of the country’s socioeconomic context.

## Author summary

Rheumatic heart disease is a serious heart condition that develops as a consequence of throat infections caused by a common type of bacteria. While this disease is preventable and now rare in many parts of the world due to better healthcare and early treatment, it continues to cause significant illness and death in Malawi, especially among children and adolescents. In this study, we reviewed existing research on rheumatic heart disease in Malawi to understand how common it is, the challenges people face accessing healthcare in the country, and what solutions may be feasible to improve the situation. We found that many patients first present to hospital when the disease is already advanced. Additionally, there is a shortage of trained healthcare workers and limited access to the medications needed to treat and prevent the disease. However, we also found that efforts to increase training for nurses and other healthcare professionals have shown promise, particularly in rural areas where resources are scarce. By highlighting these findings, our goal is to support future work to reduce the burden of this treatable disease and improve access to care in Malawi.

## Introduction

Rheumatic heart disease (RHD) is an acquired heart disease resulting from repeated infections with Group A Streptococcus (GAS), a bacterium that causes pharyngitis and cellulitis. GAS infection can trigger an autoimmune response leading to rheumatic fever (RF), which targets the valves of the heart, damaging them. If left untreated or recurrent, RF can progress to RHD, characterised by permanent valvular damage. This progression has serious implications for cardiac function, leading to heart failure and arrhythmias, and significant morbidity and mortality if left untreated [1]. The prevalence of RHD is markedly lower in high-income countries, inversely related to a country’s income status [2]. Malawi, one of the world’s poorest nations, had an estimated 323,422 RHD cases in 2021 [3,4]. The persistence of RHD in the region is attributed to a complex interplay of factors including overcrowded living conditions, inadequate sanitation, poor nutrition and limited access to healthcare and education [5–7].

In contrast to high-income countries, where early GAS infections can be readily detected using throat swabs and rapid antigen tests, diagnoses in resource-constrained settings rely primarily on clinical judgement [8]. The diagnostic role of echocardiography emerges as repeated episodes of ARF incur significant damage to the heart’s valves, causing RHD. There has been widespread adoption of portable echocardiography across Malawi since the 1990s, and its value in RHD care has been well-established in the literature from the country in the interim [9]. RHD screening programmes employ echocardiography to examine cardiac function and anatomy at the point of care and ideally detect “latent” RHD. This is an asymptomatic stage of disease in individuals with no known ARF history but with pathologic changes to the heart confirmed on echocardiography. The echocardiography findings are graded according to the World Heart Federation guidelines as ‘normal’, ‘borderline RHD’ or ‘definite RHD’ with further subdivisions according to the valvular pattern of disease [10]. In high-income countries, such screening programmes have facilitated timelier interventions and the virtual eradication of RHD. Limited access to the necessary equipment and individuals trained in their use have impeded similar progress in low-income countries.

RHD is a severe complication of treatable preceding infections. Primary prevention involves the institution of antimicrobial therapy for patients presenting with a confirmed or suspected GAS infection [1]. Prompt diagnosis is particularly important for those at risk of developing further GAS infections or ARF [11], with the greatest risk attributed to overcrowding in the household environment, increasing the risk for lower-income communities [8]. Current evidence suggests that the most effective secondary prevention strategy is the prevention of rheumatic fever with monthly intramuscular injections of benzathine penicillin G (BPG) over the course of many years [1]. The associated requirement for consistent patient monitoring poses a substantial challenge, particularly for those living in poverty, further highlighting the need to promote RHD screening and early treatment [12].

However, despite these proven preventative measures, research from Malawi suggests that the majority of RHD cases present late, with severe symptomatic disease. At this stage, patients are less likely to achieve adequate disease control with BPG injections alone [10,13]. In these communities, the challenges to treatment become increasingly difficult to surmount as the disease progresses. The definitive treatment for advanced RHD is cardiac surgery, access to which is virtually absent in most low- to middle-income countries. At present, there are no options for surgical intervention for RHD in Malawi, and cost-effective solutions and alternatives will be critical to addressing the high prevalence in the country [5].

This scoping review aims to document existing RHD research in Malawi, focusing on disease burden, barriers to care, and proposed solutions, particularly among high-risk groups like children and young adults. By mapping existing research, this study seeks to guide future studies and inform strategies to bridge healthcare disparities between high- and low-income countries in the pursuit of RHD eradication.

## Methods

A scoping review was deemed the most appropriate approach to determine the burden of GAS, ARF and RHD in Malawi and the obstacles to adequate treatment for these illnesses. Scoping reviews broadly survey the existing literature, allowing the prevailing themes and research gaps to be identified. This scoping review was designed as per the six-stage protocol outlined by Arksey and O’Malley (2005) and the PRISMA-ScR (Preferred Reporting Items for Systematic Reviews and Meta-Analyses extension for Scoping Reviews) guidelines, and was conducted as follows [14]:

1. **Identifying the research question**

Although readily treatable through primary and secondary prevention strategies, RHD continues to cause significant morbidity and mortality in many low-income countries. The objective of this scoping review is to answer the following research question: *what does the available literature tell us about the burden of RHD, ARF and GAS infections in Malawi and what barriers impede care for affected populations?*

To define the burden of GAS, ARF and RHD in Malawi, the following outcomes of interest will be investigated:

- Incidence of sore throat in children aged 5–15 years
- Prevalence of GAS among cases of sore throat
- Incidence of ARF
- Case-fatality rate from ARF
- Prevalence of RHD
- Mortality from RHD
- Prevalence of non-fatal outcomes of RHD

To establish what barriers and facilitators exist in the detection and treatment of GAS, ARF and RHD, the following will be considered:

- Initial decision to seek care
- Factors influencing diagnosis
- Factors influencing treatment or referral
- Factors influencing adherence and retention in long-term care

The role of the patient, healthcare provider and health system in each of these will be examined.

**2. Identifying relevant studies**

The preliminary literature search was conducted in July 2023 using the databases PubMed, EMBASE, Cochrane Library, and Clinicaltrials.gov. Keywords were identified and used to establish medical subheadings (MeSH) and Emtree terms in Medline and EMBASE, respectively. A number of search terms were combined to create a reading list, grouped so that eligible studies included one term from each group. A search strategy was generated and applied using a combination of terms linked to rheumatic heart disease, rheumatic fever, and pharyngitis. The search strategies are available in S1 Text.

The initial search and screening process were conducted in July 2023. Titles and abstracts were screened independently by two reviewers (EB & JG), and potentially relevant studies were selected for full-text review. Full-text screening was also performed independently by the same two reviewers. To ensure inclusion of the most recent publications, repeated searches were conducted throughout the writing process, with the most recent search performed on 5th November 2024. Any discrepancies during screening were resolved through discussion.

Filters were applied to restrict results to studies published in English, conducted in Malawi, and dated between 1995–2024. Further sources were identified by manually searching the reference lists of studies selected from the databases of interest.

**3. Study selection**

The studies chosen for inclusion in the current review satisfied the following criteria: any epidemiological study (i) focused on the individual presenting with sore throat/ARF/RHD and identifying the principal stakeholders; (ii) addressing at least one of the diseases of interest (GAS, ARF, RHD); (iii) containing one or more of the objectives outlined in 1. the research question above; (iv) conducted in or after 1995. The exclusion of research published prior to 1995 ensured that the data reflected advances in the diagnosis and care of RHD as echocardiography was only widely implemented for this purpose post-1990s [9]. Publications including participants of all ages and genders in all healthcare settings were considered. The scope of the search was restricted to literature written in English. Exclusion and inclusion criteria are outlined in Table 1.

**Table 1:** Study inclusion and exclusion criteria.

Category	Inclusion criteria	Exclusion criteria
Publication characteristics	Published 1995–2024 Published in English	Published prior to 1995 Published in languages other than English with no translation
Population	Individuals of all ages Individuals presenting with GAS, ARF or RHD	–
Study type	Journal articles, government documents, theses from Malawi	Opinion pieces, case reports, systematic reviews Case reports without broader relevance
Content Relevance	Addressed any stage of care (prevention, diagnosis, treatment, follow-up)	Studies with irrelevant focus (e.g., solely microbiological techniques without clinical application) Studies primarily discussing unrelated health services or policies Duplicate reporting of data already included from broader studies

**Fig 1:**
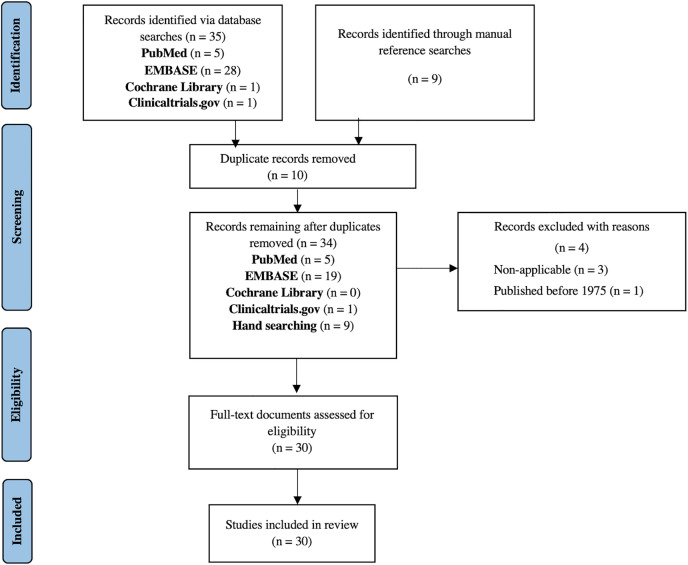
PRISMA ScR flow-chart.

The PRISMA Extension for Scoping Reviews (PRISMA-ScR) was used to determine study eligibility and to document the flow of study selection (Fig 1). In accordance with scoping review methodology, studies were included irrespective of study design, and no formal quality assessment was performed, as the objective was to map the extent and nature of the available research.

**4. Charting the data**

From the bank of relevant articles (n = 30), data extraction and charting were carried out by a single reviewer using a structured table in Microsoft Word to collate and summarise the key characteristics and findings of the included studies. The following information was charted for each study.

- First author
- Year of publication
- Literature type
- Type of study
- Area evaluated
- Main findings

**5. Collating, summarising, and reporting results**

A summary of the literature and relevant findings can be found in Supplemental Information (S2 Table). These findings are further elaborated in the results section.

**7. Consultation exercise**

The inclusion and exclusion of studies from this scoping review were guided by the advice of experts in the field of RHD in Malawi.

## Results

### Description of included studies

The PRISMA ScR flowchart (Fig 1) above outlines the approach employed for searching, identifying, and selecting the relevant records. The preliminary searches of PubMed, EMBASE, Cochrane Library and Clinicaltrials.gov yielded thirty-five records. An additional nine papers were identified by hand-searching the reference lists. Following a review of the study titles and abstracts and the removal of duplicates, thirty records met the eligibility criteria and were deemed appropriate for full-text review. At the end of this process, all thirty papers were accepted for data extraction and inclusion in the scoping review. Ten records included were conference abstracts, and seventeen were articles published in peer-reviewed journals. One clinical trial was included. The two remaining records were government policy documents included on the recommendation of an expert in the field of RHD in Malawi.

### Study design

The papers included in this review employed a wide variety of study designs. Among the thirty records included, there were six descriptive studies [6,15–19], four cross-sectional studies [20–23], four cohort studies [12,13,24,25], two pilot studies [5,26], two retrospective analyses [27,28], one observational study [29], one feasibility study [30], one concept design [31], one scoping review [32], one literature review [9], one secondary analysis [33], one external validation study [34], one interventional study [35], and one mixed-method study combining qualitative and quantitative strategies [36]. One clinical trial and two government report documents were also deemed important for inclusion in the review [37–40].

### Study population and setting

The study sample sizes varied significantly, ranging from 1,450–3,908 participants in Malawi-specific RHD screening studies [12,28]. RHD participants’ ages varied from 5-16 years [20,34] in studies from Malawi, with a female preponderance. While RHD can be diagnosed in children under 5 years of age [41], none of the included studies provided data on diagnoses in this age group, either because children under 5 were not included in the study populations or because the ages of diagnosed patients were not clearly reported. Most studies were based in urban and peri-urban settings. Seven studies were carried out in central hospitals: four in Kamuzu Central Hospital in Lilongwe [5,6,24,29], two in Queen Elizabeth Central Hospital in Blantyre [18,42], and one in Mzuzu Central Hospital [28]. Three were conducted in schools and/or nearby communities in Lilongwe [17,20,21] and Blantyre [21]. Two studies were based in rural PEN-Plus clinics, in Neno District Hospital and Lisungwi Community Hospital, offering decentralised care for RHD 110km from the nearest tertiary centre [16,35]. One study was based on Likoma Island [30].

### Outcomes of interest

#### The burden of GAS, ARF and RHD in Malawi.

The burden of GAS infection in Malawi was not formally assessed in any of the papers included in this review, although two studies reported ARF prevalence data. Zühlke et al. found that within their cohort, 22.3% of RHD patients from low-income countries had a documented episode of ARF in their medical history [19]. The data collected in this study were grouped according to the country’s economic status, and Malawi-specific figures could not be extrapolated. Another study reported that 13% of children diagnosed with RHD (n = 39) in their cohort had a history of ARF [6]. Two large multi-centre studies found that the incidence of ARF recurrence was low during the study period, irrespective of the country’s income status [29]. For instance, Zühlke et al. (2016) recorded a 0.6% incidence of ARF recurrence over a 27-month period assessing 14 low- and middle-income countries [25].

Several studies addressed the burden of RHD in Malawi according to the criteria of interest within this review including the incidence and prevalence of RHD, and the associated morbidity and mortality.

One study evaluated the incidence of subclinical RHD evolution (between normal, borderline, and definite echocardiographic categories) in Malawian schoolchildren and found that the progression or regression of subclinical disease varied substantially across participants by the end of the study period. Factors associated with changes in disease status included the pattern of valvular involvement and the use of antibiotic prophylaxis [13].

**Fig 2:**
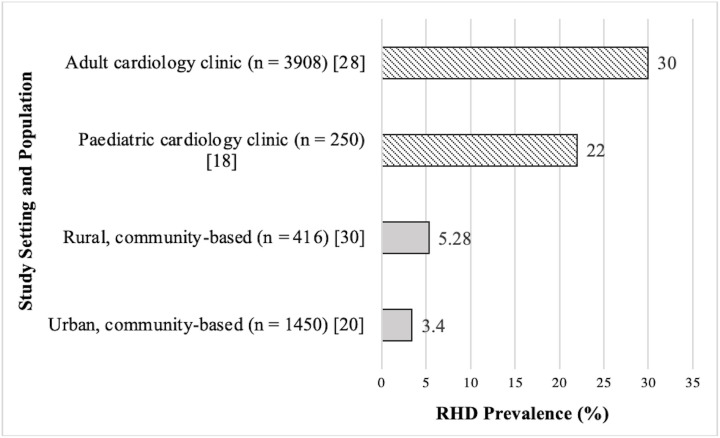
Reported RHD prevalence by study setting and population in Malawi.

Overall, six studies assessed the prevalence of RHD in Malawi: one focused on latent RHD in urban and peri-urban communities [20] and another investigated RHD prevalence in rural communities. Latent RHD was found to have a prevalence of 3.4% in schoolchildren living within 60km of the country’s capital city, Lilongwe, and the highest prevalence was among 11–13-year-olds [20]. The rural prevalence of RHD in Malawi could not be discerned from the latter study as the data from multiple low-income countries were pooled for analysis [23]. Two further studies investigated RHD prevalence in cardiology clinics in Malawi, one in a paediatric population and the other in adult patients. 22% of the children (n = 250) and 30% of the adults (n = 3908) presenting to the respective cardiology clinics had manifest RHD [28]. In another study based on Likoma Island in Lake Malawi, 5.28% of the children who underwent echocardiographic screening (n = 416) had RHD [30]. These findings are summarised visually in Fig 2.

A number of studies investigated the outcomes of RHD, in terms of mortality and morbidity. Fatal outcomes were evaluated in three studies [15,25,29]. One study reported that 0.6% of inpatient deaths in a large tertiary hospital in Malawi were attributable to RHD or rheumatic fever, while the leading causes of death were sepsis and lower respiratory tract infections [15]. Two additional studies combined data from several low-income countries for analysis, but Malawi-specific data were not independently reported [25,29]. Across these two studies, patient factors including increasing age, lower income status, poorer education and more severe valvular disease were associated with increased RHD mortality [25,29]. Karthikeyan et. al (2024) reported that, across income groups, heart failure and sudden cardiac death accounted for most RHD-associated deaths [29]. Mortality was lower among females, participants with an education beyond primary school, those with a history of valvuloplasty or valve surgery, and those using secondary antibiotic prophylaxis. While a smaller proportion of patients from low-income countries underwent valve surgery, the use of secondary antibiotic prophylaxis was higher in this cohort compared to those in middle-income countries [29].

Non-fatal outcomes and complications of RHD were investigated in four studies. The adverse clinical outcomes described included congestive heart failure (CHF), stroke, atrial fibrillation, infective endocarditis, and worsening echocardiographic RHD status [19,25,29,34].

One study of RHD across multiple countries found that more patients in low-income countries were diagnosed with heart failure, both at baseline and the two-year follow-up, compared to those in upper-middle-income countries. Conversely, stroke and infective endocarditis were more prevalent in upper-middle-income groups [25]. Another, more recent study that included Malawi reported that the incidence of stroke, infective endocarditis, and recurrent rheumatic fever was low across income groups during the three-year follow-up period. Supporting earlier findings, this study also found that the highest risk of stroke was in the upper-middle income group [29].

Patterns of disease evolution were evaluated in two studies [12,13]. Four studies evaluated the characteristics of RHD, highlighting the extent of severe forms of the disease [6,21,28,43]. Another two documented patient characteristics as well as complications of the disease [19,25]. Of note, a significant number of patients with RHD in Malawi present to healthcare late with severe disease. Severe first presentations were reported between 62%-82.5% across three separate studies [6,17,21].

### Barriers to care

#### Factors influencing the initial decision to seek care.

Within the studies included in this review, there was limited information about the factors influencing an individual’s initial decision to seek care. The available research consistently reiterates the observation that RHD patients in Malawi present to healthcare with advanced disease [6]. This is attributed, in part, to inadequate education about the dangers associated with repeated ARF episodes and the logistical obstacles to healthcare access.

The PEN-Plus clinics established in rural Neno have been instrumental, particularly for those living remotely, by reducing the need for long-distance travel and improving access to healthcare for the poorest communities in Malawi. These clinics are run by mid-level providers trained to diagnose RHD, implement early treatment plans, and refer to specialists where appropriate. Additionally, community health workers visit patients who miss follow-up clinics to identify and address barriers that may exist for the individual and to re-enrol them in care [16].

#### Factors influencing diagnosis.

Many studies found that inadequate RHD-specific education for Malawian communities and healthcare workers poses a significant challenge to RHD diagnosis and management in the country. Recent research from Malawi is largely focused on the assessment of RHD education in the country and the implementation of programmes aimed at improving awareness.

Eight studies in this review investigated RHD education strategies, with an emphasis on training non-physician providers to diagnose and treat RHD [5,16,22,26,27,30,32,35]. This approach, frequently referred to as “task-shifting”, emerges as a salient theme across the literature. There was a consensus across these studies that training nurses and clinical officers, collectively termed mid-level providers, was a feasible and cost-effective means for improving RHD diagnosis in Malawi.

The structure of the education programmes varied across the different studies with courses spanning from several days to months and combining didactic lectures, supervised ultrasound training including image interpretation alongside clinical mentoring [26,27,35]. Trainees were at various qualification levels and included medical students, general physicians, clinical officers, nurses, and physician assistants. The number of trainees ranged from two to sixty-five [5,30].

The evaluation of training efficacy also differed among these studies. Three studies examined the sensitivity and specificity of RHD diagnosis after training, finding reasonable congruence between trainees’ and cardiologist diagnoses [22,26]. Two studies conducted pre- and post-training assessments, with retention assessments one year and six months later, respectively. At six months, test scores remained high at an average of 84.4%, compared to a pre-test average of 63.3% and an immediate post-test average of 88.3%. In another study, average test scores one year after training had fallen to 69.5% from 83.2% in the immediate post-training period, highlighting a need for ongoing training and re-assessment to ensure that adequate standards are maintained among these providers [27,35]. Another study employed a post-workshop questionnaire.

One study assessed the standard of knowledge among healthcare providers regarding the prevention of ARF and RHD in a central hospital. Participating nurses and clinicians reported concerns about the safety of BPG for RHD prevention. A subsequent RHD education workshop improved participants’ understanding of and comfort with prescribing the medication [5].

#### Factors influencing treatment and adherence.

Many studies in this review reveal that inadequate resources, in the form of medication, medical equipment and human power, impede RHD care in Malawi in a number of ways [12,19,29,33]. For instance, one study identified resource limitations as a key barrier to treatment adherence. After the first year of the study, the healthcare centres treating the participants did not have a steady supply of BPG. Therefore, many children did not receive their injections despite presenting for their appointments. In light of this, BPG was provided directly to the participants for the following year which improved adherence but revealed a further barrier to treatment; an insufficient supply of the syringes required for BPG administration at the healthcare centres [12]. A large multi-centre study similarly found that a significant proportion of RHD patients in low-income countries (up to 50%) received suboptimal secondary RHD prophylaxis [19].

Alongside medication and equipment shortages in Malawi, the workforce is insufficiently powered to facilitate long-term follow-up and surgical interventions for many RHD patients. Follow-up is critical to ensure appropriate RHD prophylaxis, alongside monitoring for and control of RHD complications. As mentioned, “task-shifting” is likely to play a central role in expanding the effective workforce to improve longitudinal RHD care. Unfortunately, the lack of cardiac surgeons in the country also poses an issue. The REMEDY study revealed that valvular repair and replacement were positively correlated with a country’s income level [19]. In this study population, there were twice as many valve surgeries carried out in high-income- relative to low-income-countries, despite a higher prevalence of RHD cases requiring surgical intervention in the latter. This finding was reiterated in a more recent multi-centre study, which reported that 3.3% of RHD patients across low-income countries underwent valve surgery compared to 7.2% of those from upper-middle-income countries included in the study. Karthikeyan et al. suggest that referring patients with advanced RHD to lower-middle-income countries like India for valve surgery may be more cost-effective than developing in-country surgical infrastructure in low-income countries like Malawi [29].

#### Factors influencing retention in long-term care.

The factors influencing retention in long-term care were not elucidated in the studies in this review. An interplay between the prolonged nature of RHD treatment and various socio-economic factors is likely. One research group showed that by utilising established patient tracking systems in Malawi, such as the HIV-defaulter system, they could maximise retention in care throughout the study period, providing a possible solution to the issue of long-term follow-up of RHD patients in Malawi [12]. Table 2 provides a summary of the various barriers to RHD care identified across the included studies.

**Table 2:** Key barriers to RHD care in Malawi identified across included studies.

Barrier Category	Description	Studies reporting
Inadequate education & training	Need for standardised, ongoing education efforts for healthcare providers and improved public awareness of RHD.	[5,16,22,26,27,30,32,35]
Medication & equipment shortages	Unreliable supply of BPG and healthcare equipment required for diagnosis and treatment.	[12,19,33]
Human resource limitations	Limited workforce trained to diagnose, treat, and follow-up patients with RHD.	[12,19]
Access to surgical care	Minimal in-country surgical infrastructure; disparity in surgery rates between income groups.	[19,29]
Geographic & infrastructural barriers	Long travel distances to care facilities; poor rural access; mitigated in some regions by PEN-PLUS clinics.	[16]
Long-term retention in care	Socio-economic and structural barriers to long-term follow-up.	[12]

## Discussion

This scoping review outlines the current literature on the burden of rheumatic heart disease in Malawi, identifies barriers to RHD care, and highlights the proposed solutions. In 2015, RHD experts, in collaboration with the Social Cluster of the African Union Commission, developed a list of strategies to eliminate ARF and RHD in Africa. In Malawi, researchers have increasingly evaluated these strategies, including the decentralisation of diagnostic expertise and the creation of disease registers. Despite this progress, Malawi still faces numerous challenges. Overcoming them will require a better understanding of the barriers to care for individuals and communities, a consistent penicillin supply, the development of cardiac surgery centres, and collaboration with international corporations to facilitate resource mobilisation, monitoring, and programme evaluation [44].

### Key findings

#### Screening and diagnosis.

The optimisation of RHD echo-based screening and diagnosis in Malawi is a prominent theme in this review. Screening programmes have not only provided valuable diagnostic insights but also helped quantify the national burden of RHD [3,5]. However, widespread screening in endemic regions raises concerns about the potential over-treatment of latent RHD. A recent randomised controlled trial demonstrated a similar regression rate of latent RHD in both BPG-treated and placebo groups, suggesting some patients may undergo unnecessary treatment [45]. The use of simplified echocardiographic scoring systems could help stratify patients by risk and enable more targeted medication use, especially in settings with limited resources [34].

#### Rural health disparities.

A significant gap in the literature concerns the prevalence of RHD in rural communities, which account for 84% of Malawi’s population. Most reviewed studies are based in urban or peri-urban settings, and many in the country’s largest hospitals [19,28]. The research to date, therefore, likely excludes large portions of the population who lack access to tertiary care [28,37]. Research from other Sub-Saharan countries suggests that rural communities experience higher RHD prevalence, indicating Malawi may be underestimating its rural disease burden, possibly complicating eradication efforts [46,47].

#### Antecedent illnesses research.

The research provides limited insight into the preceding illnesses of RHD, namely GAS and ARF. While the relevant terminology may not have been captured in the search strategy, this finding may also reflect health-seeking behaviours in Malawi. Many individuals may avoid or fail to access formal care during early infections, meaning these cases go undocumented in facility-based research. Community-level studies could clarify how individuals make decisions about seeking care and enhance our understanding of early barriers to care [6]. Furthermore, evaluating the feasibility of primordial prevention - strategies that minimise the risk of disease before it begins - will be crucial. Despite widespread recognition of the link between poor social conditions and GAS/ARF development, little is known about the impact that changes at this level may have on disease prevalence and trajectory in the resource-poor communities most susceptible to RHD [19].

#### RHD-specific education for Malawian healthcare workers.

There has been a notable shift in the focus of RHD research from Malawi in recent years. Earlier studies laid crucial foundations by mapping disease patterns and highlighting the significant burden of disease. More recent efforts have shifted towards addressing the shortage of physicians trained in RHD care. Task-shifting programmes, which educate non-physician healthcare workers in the diagnosis and treatment of RHD, offer promise. PEN-Plus clinics in Malawi currently use this model, and similar approaches have succeeded elsewhere, including in rural Rwanda, by reducing the need for specialist cardiology input in resource-limited settings [16,48].

#### RHD-specific education for Malawian communities.

Despite the high disease burden, the literature lacks studies exploring community-focused RHD education. Although the search strategy may have missed relevant sources, this may reflect a genuine gap in the literature. In the absence of effective community education, the link between GAS-pharyngitis and long-term RHD complications may not be widely known, resulting in many individuals progressing from subclinical to manifest disease before seeking care.

#### Retention in care.

Long-term retention in RHD care presents a substantial challenge in Malawi due to multiple interacting factors. Shortages of medication and medical equipment, coupled with lower education status, and long travel distances to healthcare facilities hinder consistent engagement with healthcare services [19,25,49,50]. A large multi-centre study reported an 11.5% loss to follow-up, especially among patients with severe disease and lower education status, underscoring the barriers to the continuity of care in low-resource settings [25].

Although alternative treatments that reduce the need for frequent hospital visits remain unexamined, community-based approaches offer hope. For instance, PEN-Plus clinics have trained community health workers to visit patients missing appointments and provide social support. This intervention has improved engagement significantly and a roll-out of these strategies across the country could considerably improve retention in care, improving patient outcomes and reducing the RHD burden [16].

## Strengths & limitations

By utilising the Arksey and O’Malley framework, this scoping review mapped the available RHD literature from Malawi according to a rigorous and standardised approach for study selection and analysis. Nonetheless, the scoping review process inherently lacks an assessment of study quality, instead outlining all relevant resources regardless of quality, unlike a systematic review. Additionally, the literature search was limited to articles written in or translated into English and the results of this scoping review are unlikely to represent the full repertoire of research available on the topic of RHD in Malawi. Finally, a number of studies included Malawian participants as part of a low-income group without presenting each country’s data separately, thus allowing only generalised deductions to be made in these instances.

## Comparisons with the existing literature

We identified one other scoping review detailing RHD research in Malawi during our study selection process. Our findings build upon those of Zhao et. al, who mapped the literature on task-sharing in Malawi. Since their publication, further studies have been published on task-sharing, reinforcing the importance of maximising the country’s available human resources in the efforts to eradicate RHD. By mapping the current research landscape, our review complements the existing literature, highlights prevailing themes, and identifies gaps to guide future research in a meaningful way.

## Implications of this review

The findings of this scoping review indicate that the burden of RHD and preceding illnesses remains high in Malawi despite the availability of cost-effective screening and treatment. Multiple contributing factors drive this ongoing burden. Currently, Malawi has only one paediatric cardiologist and no in-country cardiac surgeons, despite a population of 22 million and a significant burden of cardiac disease [16]. Additional issues include failures to recognise and manage GAS pharyngitis and ARF in primary care [51], insufficient education for communities and healthcare workers regarding prevention, a high prevalence of asymptomatic disease that delays health-seeking behaviours, and barriers to care such as the need for repeated BPG injections and long-term monitoring.

## Conclusion

In Malawi, and throughout Sub-Saharan Africa, RHD remains a leading cause of morbidity and mortality despite successful eradication efforts in high-income countries [52]. If the WHO is to achieve its goal of a 25% reduction in mortality from rheumatic fever and RHD in under 25s by the year 2025, future research from Malawi, where the burden is significant, must provide direction to that end [53]. Resource scarcity and additional barriers to care must be considered in the solution. The questions to be addressed are why does RHD persist in these communities and what solutions are realistic in the socio-economic setting of countries like Malawi? The answers will guide population-specific RHD care, inform government policy and facilitate appropriate resource allocation, with the goal of reducing the significant health inequity between high- and low-income countries, such as Malawi.

## Supporting information

S1 Text Search strategies. Full search strategies used within PubMed, Embase, and Cochrane Library databases to identify relevant studies for the scoping review.
(DOCX)

S1 Table Study inclusion and exclusion criteria. Summary of the predefined inclusion and exclusion criteria applied throughout screening and selection of sources for the scoping review, presented across four domains: publication characteristics, population, study type, and content relevance. GAS: *Group A Streptococcus*; ARF: *acute rheumatic fever*; RHD: *rheumatic heart disease*.
(PDF)

S2 Table Description of studies included. Detailed characteristics of all studies included in the scoping review, including study design and key findings.
(PDF)

S1 Fig PRISMA ScR flow-chart. Flow diagram illustrating the identification, screening, eligibility assessment, and inclusion of sources in accordance with the PRISMA-ScR (Preferred Reporting Items for Systematic reviews and Meta-Analyses extension for Scoping Reviews) guidelines.
(TIF)

S2 Fig Reported RHD prevalence by study setting and population in Malawi. Prevalence of rheumatic heart disease (RHD) (%) reported across four study settings: adult cardiology clinic, paediatric cardiology clinic, rural community-based, and urban community-based populations. Sample sizes (n) and reference numbers correspond to the included studies.
(TIF)

S1 PRISMA Checklist PRISMA-ScR Checklist. Completed PRISMA-ScR (Preferred Reporting Items for Systematic reviews and Meta-Analyses extension for Scoping Reviews) checklist for this study.
(DOCX)
