# Supplementary material for: The burden of rheumatic heart disease and issues affecting the provision of care in Malawi: A scoping review
Source: PLoS Negl Trop Dis. 2025 Aug 19;19(8):e0013400. doi: 10.1371/journal.pntd.0013400 (PMC12380299; doi:10.1371/journal.pntd.0013400)
Supplement: S1 Text — Full search strategies used within PubMed, Embase, and Cochrane Library databases to identify relevant studies for the scoping review. (DOCX) [file pntd.0013400.s001.docx]

**S1. Search strategies**

**PubMed search strategy: performed 05/11/2024**

("Rheumatic Heart Disease"[MeSH Terms] OR "rheumatic heart disease*"[Title/Abstract] OR "rheumatic heart"[Title/Abstract] OR "rheumatic carditis"[Title/Abstract] OR

("Rheumatic Fever"[MeSH Terms] OR "rheumatic fever*"[Title/Abstract] OR "acute rheumatic fever*"[Title/Abstract]) OR

("Streptococcus pyogenes"[MeSH Terms:noexp] OR "Pharyngitis"[MeSH Terms] OR "Group A streptococcus"[Title/Abstract] OR "group a streptococcal infection*"[Title/Abstract]))

AND

("Cost of Illness"[MeSH Terms] OR "Morbidity"[MeSH Terms] OR "Mortality"[MeSH Terms] OR "Prevalence"[MeSH Terms] OR "Incidence"[MeSH Terms] OR

(("Diagnosis"[MeSH Terms] OR "Referral and Consultation"[MeSH Terms] OR "Therapeutics"[MeSH Terms] OR "Treatment Outcome"[MeSH Terms] OR

"Treatment Refusal"[MeSH Terms] OR "Stakeholder Participation"[MeSH Terms] OR "Patient Compliance"[MeSH Terms] OR "Health Resources"[MeSH Terms] OR

"Resource Allocation"[MeSH Terms] OR "Resource-Limited Settings"[MeSH Terms] OR "Delivery of Health Care"[MeSH Terms] OR

"health care quality, access, and evaluation"[MeSH Terms] OR "Healthcare Disparities"[MeSH Terms] OR "Health Workforce"[MeSH Terms] OR

"Health Policy"[MeSH Terms] OR "Continuity of Patient Care"[MeSH Terms])

AND "Health Communication"[MeSH Terms]) OR "Health Facility Administration"[MeSH Terms] OR "Social Determinants of Health"[MeSH Terms])

AND ("Malawi"[MeSH Terms] OR "malawi*"[Title/Abstract])

**Embase search strategy: performed 05/11/2024**

('malawi'/mj OR 'malawi*':ti,ab,kw)

AND

('rheumatic heart disease'/mj OR 'rheumatic carditis'/exp OR 'rheumatic heart disease*':ti,ab,kw OR 'rheumatic heart':ti,ab,kw OR 'rheumatic carditis':ti,ab,kw OR

('rheumatic fever'/exp OR 'rheumatic fever*':ti,ab,kw OR 'acute rheumatic fever*':ti,ab,kw) OR

'streptococcal pharyngitis'/syn OR 'group a streptococcal infection'/de OR 'group a streptococcus':ti,ab,kw OR 'group a streptococcal infection*':ti,ab,kw)

AND

('disease burden'/syn OR 'morbidity'/syn OR 'mortality'/syn OR 'prevalence'/de OR 'incidence'/de OR 'diagnosis'/syn OR 'patient referral'/exp OR 'therapy'/syn OR

'treatment outcome'/syn OR 'treatment refusal'/de OR 'patient compliance'/exp OR 'stakeholder engagement'/syn OR 'resource limited setting'/syn OR

'health care concepts'/exp OR 'health workforce'/syn OR 'health care policy'/de OR 'patient care'/syn OR 'hospital management'/syn OR 'social determinants of health'/syn)

**Cochrane Library Reviews search strategy: performed 05/11/2024**

(rheumatic* OR "group A streptococcus" OR "rheumatic heart disease" OR "rheumatic fever" OR "acute rheumatic fever" OR "pharyngitis")

AND

("cost of illness" OR morbidity OR mortality OR prevalence OR incidence OR "healthcare access" OR "health care delivery" OR "health care quality" OR "resource allocation" OR "health care disparities" OR "health workforce" OR "patient compliance" OR "health policy" OR "social determinants of health")

AND

Malawi
