# Supplementary material for: The burden of rheumatic heart disease and issues affecting the provision of care in Malawi: A scoping review
Source: PLoS Negl Trop Dis. 2025 Aug 19;19(8):e0013400. doi: 10.1371/journal.pntd.0013400 (PMC12380299; doi:10.1371/journal.pntd.0013400)
Supplement: S1 Table — Summary of the predefined inclusion and exclusion criteria applied throughout screening and selection of sources for the scoping review, presented across four domains: publication characteristics, population, study type, and content relevance. GAS: Group A Streptococcus; ARF: acute rheumatic fever; RHD: rheumatic heart disease. (PDF) [file pntd.0013400.s002.pdf]

S2 Table. Description of studies included.

Author, year	Study title	Literature type	Study design	Area evaluated	Major/relevant findings
Sanyahumbi et. al, 2019 (12)	***Two-year evolution of latent rheumatic heart disease In Malawi***	Journal article: *Congenital heart disease*	Cohort study	Evaluating the progression/regression of RHD in Malawian schoolchildren diagnosed through echocardiogram screening.	• Patients screened (n = 1450)<br>• Patients with diagnosed RHD (n = 50)<br>• Risk of borderline progression was low<br>• 39 had borderline RHD:<br>- 48.7% remained borderline, 2.6% progressed to definite, 43.6% regressed to normal, 2.6% were reclassified, 2.6% were lost to follow-up<br>• 11 had definite RHD:<br>- 54.5% remained definite, 36.4% regressed to borderline, 9.1% regressed to normal

					• Penicillin adherence was suboptimal<br>- Only 2/11 (18.2%) with definite RHD had penicillin adherence >80% for the 2-year follow-up period.<br>- Inadequate resources in the healthcare centres contributed to poorer adherence<br>• No significant differences in measured characteristics between those with definite RHD that regressed to borderline and those who did not.
Sanyahumbi et. al, 2017 (26)	***Task shifting to clinical officer-led echocardiography screening for detecting***	Journal article: *Cardiology in the young*	Pilot study	Training clinical-officers over five days in echo-based RHD screening with subsequent evaluation of performance relative to trained paediatric cardiologists.	• Clinical officer trained (n = 8)<br>• There was considerable agreement between clinical officers and paediatric cardiologists about whether to refer

	rheumatic heart disease in Malawi, Africa				• Trained clinical officers had high sensitivity (91%) and acceptable specificity (65%) for latent RHD detection according to the World Heart Federation criteria.
Harris et. al, 2019 (15)	***Paediatric deaths in a tertiary government hospital setting, Malawi***	Journal article: *Paediatrics & International Child Health*	Prospective, descriptive study	Determining the causes of inpatient child deaths in Queen Elizabeth Central Hospital, Malawi over a 12 month period.	• Total paediatric admissions (n = 13, 827)<br>• Total deaths (n = 488 deaths)<br>• Deaths caused by RHD/rheumatic fever (n = 3; 0.6%)
Sanyahumbi et. al, 2016 (20)	***School and Community Screening Shows Malawi, Africa, to Have a High Prevalence of Latent Rheumatic Heart Disease***	Journal article: *Congenital heart disease*	Cross-sectional study	Determining the prevalence of latent RHD among children aged 5-16 in Lilongwe, Malawi through echocardiographic screening between February and April 2014.	• Participants screened (n= 1450) across three schools and the surrounding communities in Lilongwe, Malawi.<br>• The prevalence of latent RHD was 3.4%<br>- 0.7% definite RHD<br>- 2.7% borderline RHD

					• There were no significant differences in RHD prevalence based on gender, location (urban vs. peri-urban), or age.
Puri et. al, 2018 (24)	***Pattern of inpatient pediatric cardiology consultations in sub-Saharan Africa***	Journal Article: *Congenital heart disease*	Cohort study	Review of demographic, anthropometric, and clinical information for all cardiology consults admitted to the children's wards in Kamuzu Central Hospital in Lilongwe, Malawi over a 1 month period.	• Total admissions (n = 1623)<br>• Cardiology consults (n = 73)<br>- 6 (8.2%) had a history of RHD<br>• Echocardiograms performed (n = 69)<br>- 74% were abnormal<br>- 10.1% had rheumatic heart disease with preserved cardiac function<br>• 20.3% had symptomatic systolic heart failure with reduced ejection fraction<br>• Of those who had had a previous echocardiogram (n = 20), 10% were done by a cardiologist

					• 3 patients left the hospital against medical advice<br>• Overall admission mortality was 5.5%
Author, year	Study title	Literature type	Study design	Area evaluated	Major/relevant findings
Kwan et al., 2023 (35)	***Decentralizing heart failure diagnosis and management using a task-shifting training approach in rural Rwanda, Malawi and Liberia***	Conference abstract: ***Journal of the American College of Cardiology***	Interventional study (pre-post)	Healthcare providers in three low-income countries were trained to diagnose common cardiac conditions in rural PEN-Plus clinics over 1-2 months. All participants' knowledge was assessed before and after training. A retention test was carried out 1 year later.	• Trainees included general physicians, nurses, clinical officers (CO) and physician assistants (PA) involved in chronic disease care.<br>• Five trainees from Malawi were included but results from Malawian participants were not reported separately.<br>• Pre- vs. post-test scores:<br>- 46% vs. 77.3% for all trainees<br>- 51.8% vs. 84.6% for physicians<br>- 38.5% vs. 71.8% for nurses<br>- 61% vs. 83.2% for COs and the PA.

					• Retention test scores (for COs and PA):<br>- Mean test score of 69.5%
Mailosi et. Al, 2023(16)	***Decentralized Heart Failure Management in Neno, Malawi***	Journal article: *Global Heart*	Descriptive study	A descriptive study outlining the clinical characteristics, disease categories and outcomes for patients diagnosed with HF by non-physician providers at PEN-Plus (Package of Essential NCD Interventions) clinics in Neno, Malawi.	• Non-physician providers trained in focused cardiac ultrasound (FOCUS) can improve cardiac care for patients in rural Malawi.<br>• RHD is one of the commonest causes of HF in rural Malawi, after hypertensive heart disease and cardiomyopathy.<br>• Patients with HF (n = 178)<br>• Echocardiographic determination of HF cause (n = 154)<br>- 7.1% had RHD, 26% cardiomyopathy and 36.4% hypertensive heart disease

					• Only 36% of the RHD patients were < 18 years old, suggesting late progression to HF.<br>• The study achieved high retention rates (78%) aided by transport reimbursement, and home visits for those absent from follow-up appointments<br>• 12% were lost to follow-up<br>• Many HF symptoms were reduced at follow-up suggesting effectiveness and feasibility of mid-level provider training for cardiac care using the FOCUS tool in rural Malawi.

Ruderman et al., 2022(27)	***Training Mid-Level Providers to Treat Severe Non-Communicable Diseases in Neno, Malawi through PEN-Plus Strategies***	Journal article: *Annals of Global Health*	Retrospective analysis	Training mid-level providers in PEN-Plus clinics in Neno, Malawi and assessing the impact of these clinics by evaluating healthcare provider knowledge, patient recruitment and care provision.	• Training involved seven days of didactic lectures, case studies and patient counselling teaching followed by longitudinal onsite mentoring<br>• Clinical knowledge was evaluated through a 40-point questionnaire immediately before and after training, and at 6 months after training.<br>• There was a significant improvement in provider test scores immediately following didactic training and after 6 months<br>• Averages scores:<br>- Pre-test: 63.3%<br>- Post-test: 88.3%<br>- At 6 months: 84.4%

					• Over 350 patients were enrolled in the first 18 months of the PEN-Plus clinic<br>• The clinics significantly improved the provision of medications and testing across a range of services.
Zhao et. al, 2021(32)	***Task-sharing to support paediatric and child health service delivery in low- and middle-income countries: current practice and a scoping review of emerging opportunities***	Journal article: *Human resources for health*	Scoping review	Investigating current practice and emerging opportunities for task-sharing in five African countries, including Malawi, to aid paediatric care of complex/chronic conditions including RHD.	• The scoping review identified 14 studies from Malawi pertaining to task-sharing in healthcare.<br>- 3 studies specifically evaluated task-shifting for RHD screening and management in Malawi.

Abdullahi et al., 2021(36)	***The RHD Action Small Grants Programme: Small Investment, Big Return!***	Journal article: *Global heart*	Mixed method study	Evaluates the impact and effectiveness of the RHD Action Small Grants Programme in recipient countries, including Malawi.	• This study used phone interviews and online surveys to assess the impact of the grant programme on RHD care.<br>• Modest monetary investment in the form of grants can support RHD activism and education in Malawi.<br>• Two proposals from Malawi were funded by the RHD Action Small Grants Programme<br>- In 2017, ARF and RHD workshops were given in Lilongwe, Malawi for doctors, nurses and allied health professionals (n = 65)<br>- In 2019, an RHD Awareness campaign was funded for people living with RHD and community (n = 357)

Author, year	Study title	Literature type	Study design	Area evaluated	• Major/relevant findings
Nascimento et al., 2021(34)	***Outcomes of Echocardiography-Detected Rheumatic Heart Disease: Validating a Simplified Score in Cohorts From Different Countries***	Journal article: *Journal of the American Heart Association*	External validation study	Simplification of an echocardiographic risk score for predicting RHD outcome for application in latent RHD populations in Malawi, New Zealand, Australia and Brazil.	• Malawi (n = 40)<br>• At baseline, Malawian participants had one of the highest rates of definite RHD (27.5%) in the cohort, after New Zealand (31.9%)<br>• The study established a simplified score based on the WHF criteria for latent RHD<br>• Children (age 5-16) were stratified into low (25%), intermediate (52.5%) and high (22.5%) risk groups and the 3 year risk of unfavourable outcomes was estimated using the score<br>• 20% of the Malawi cohort developed unfavourable outcomes over the 3

					years, which were defined as echocardiographic progression to a more severe diagnostic category, remaining with definite RHD, or worsening grade of valvular pathology<br>• The score model showed a significant association with unfavourable disease outcomes
Gupta et. al, 2020(33)	***Availability of equipment and medications for non-communicable diseases and injuries at public first-referral level hospitals: a cross-***	Journal article: *BMJ open*	Secondary analysis	Assessing the availability of essential equipment and medications required for a range of acute and chronic conditions in eight low-income countries including Malawi.	• Total first referral-hospitals surveyed (n = 797)<br>• Facilities in Malawi (n = 43) included public rural and community hospitals<br>• Equipment and medications considered necessary for RHD care were; essential HF medications and equipment,

	sectional analysis of service provision assessments in eight low-income countries				benzathine penicillin injections, oral penicillin and injectable epinephrine.<br>• HF medications and equipment were available in 12%, benzathine penicillin in 100% and epinephrine in 88% of the hospitals surveyed in Malawi.<br>• Data was not available for oral penicillin.<br>• Ultrasound equipment for diagnosis of RHD and HF was reported in only 51% of the facilities.<br>• Only 9% of the facilities in Malawi had all of the necessary equipment and medication for management of RHD.<br>• Self-reported availability of medications and equipment was generally much higher than the observed availability.

Olsen et al, 2020(21)	***Characteristics of rheumatic heart disease among children in Malawi***	Conference abstract: ***World Journal for Pediatric and Congenital Heart Surgery***	Cross-sectional study	Characterisation of RHD in schoolchildren using echocardiography in Lilongwe and Blantyre, Malawi.	• Patients screened (n = 124)<br>- 64.5% female<br>- 50.0% ≥ 14 years old<br>• Mitral regurgitation was the most common valvular pathology (92.7%)<br>• Mitral stenosis (84.7%), tricuspid regurgitation (84.6%) and aortic regurgitation (50%) followed in prevalence.<br>• 50.8% had both mitral and aortic valve involvement.<br>• Functional capacity was relatively preserved<br>• For patients with available diagnostic echo reports (n = 80), 82.5% had initially presented with severe RHD.

Sanyahumbi et al, 2019(13)	***Evolution of subclinical rheumatic heart disease: A multi-centre retrospective cohort study***	Conference abstract: ***European Heart Journal***	Cohort study	Determining the incidence of and factors associated with progression and regression of RHD in children with latent disease across multiple countries and regions, including Malawi.	• Children (n = 482)<br>• Children with definite RHD (n = 131)<br>• 48 (37%) regressed to borderline/normal<br>• 83 (63%) remained definite<br>• Children with borderline RHD (n = 351)<br>• 39 (11.1%) progressed,<br>• 156 (44.4%) remained borderline<br>• 156 (44.4%) regressed to normal<br>• Univariate analysis revealed that good adherence (>80%) to penicillin prophylaxis (BPG) was associated with more regression among all patients (definite + borderline)<br>• This association did not remain significant after adjustment.

					• With multivariable analysis, borderlines prescribed BPG was the only factor related to progression from borderline to definite
Sanyahumbi, Chiromo and Chiume, 2019(5)	***Education: The prevention of acute rheumatic fever and rheumatic heart disease in Malawi***	Journal Article: *Malawi medical journal: the journal of Medical Association of Malawi*	Pilot study	A pilot RHD education programme for healthcare providers in Kamuzu Central Hospital in Lilongwe, Malawi.	• Health providers (n = 65): 51 nurses, 3 doctors, 9 COs, 2 unspecified<br>• An ARF/RHD curriculum covered early stages of disease, complications, administration and safety of benzathine penicillin and RHD in Malawi<br>• Three half-day workshops<br>• Pre-workshop questionnaires revealed nurses concerns about the safety of benzathine penicillin<br>• Post-workshop questionnaires revealed that participants were much more

					comfortable prescribing/injecting benzathine penicillin after the workshop<br>• Pre-test knowledge scores improved from 43.8% to 78.5%
Author, year	Study title	Literature type	Study design	Area evaluated	Major/relevant findings
Sanyahumbi et. al, 2018(6)	***Rheumatic Heart Disease With Late Presentation Among Children In Malawi***	**Conference abstract: *Global Heart***	Descriptive study	Evaluate the characteristics of children with RHD attending the paediatric cardiology clinic at Kamuzu Central Hospital in Lilongwe, Malawi.	• The majority of RHD cases in Lilongwe present late.<br>• Children with RHD (n = 39)<br>• At presentation:<br>• 82% had severe RHD<br>• 72% had NYHA class 4 symptoms.<br>• 26 (67%) were females<br>• Age range of 6 to 17 years<br>• At initial diagnosis<br>• 1 (3%) had mild<br>• 6 (15%) had moderate

					• 32 (82%) had severe RHD<br>• 5 (13%) had a previous history of ARF<br>• 4 (10%) had had mitral valve replacements.<br>• On the most recent echocardiogram<br>• 4 (10%) had no evidence of RHD<br>• 3 (8%) had mild RHD<br>• 3 (8%) had moderate RHD<br>• 29 (74%) had severe RHD.

Hardie et al, 2016(30)	***Feasibility of focused cardiac ultrasound by non-expert operators as a strategy for a rheumatic heart disease screening programme***	Conference Abstract: ***European Heart Journal***	Feasibility study	Two medical students had one hour of training in portable cardiac ultrasound on Likoma Island, Malawi.	• Children (n = 416), aged 9-12<br>• Medical students classified ultrasound images and clinical examinations as suspicious or non-suspicious for RHD (according to 2012 World Heart Federation criteria)<br>• Suspicious cases were re-scanned by a cardiologist<br>• All images were blindly reviewed by two experts<br>**<u>Echocardiography</u>**<br>• Prevalence of RHD confirmed by expert cardiac ultrasound was 5.28%<br>• Medical students reported 26 suspicious cases<br>• Expert re-scanning identified

					• 17 true positives<br>• 5 false negatives<br>• 21 cases of inter-observer variability<br>• Sensitivity: 77.27%<br>• Specificity: 97.58%,<br>• Positive predictive value: 65.38%<br>• Negative predictive value: 98.64%.<br>**Auscultation**<br>• Auscultation revealed<br>• 7 true positives<br>• 45 false positive cases<br>• 349 true negatives<br>• 15 false negatives.<br>• Compared with expert image review, sensitivity was 31.82% and specificity 88.59%.

Sanyahumbi et. al, 2016(31)	***The concept and design of definerhd: A study to evaluate the progression of subclinical rheumatic valve lesions diagnosed through echocardiographic screening***	Conference Abstract: ***Global Heart***	Concept design	Enrolling cases of subclinical RHD to DefineRHD,, a registry to describe the natural history of subclinical RHD and the effect of benzathine penicillin G (BPG) on subclinical lesions. The data collected will include echocardiograms which will be read for standardised diagnosis as well as BPG adherence data.	• This is a concept study that will be useful in evaluating the potential benefit of widespread RHD echo screening.
Sims et. al, 2015(22)	***Clinical-officer led echocardiographic screening is sensitive for diagnosing rheumatic heart disease in Malawi, Africa***	Conference abstract: *Circulation*	Cross-sectional study	Evaluating trained clinical-officer-led diagnosis of RHD using echo compared with paediatric cardiologists. 8 COs were trained over three days in the use of portable echocardiograms for RHD screening.	• The mean kappa statistic comparing clinical-officer echo reads to the paediatric cardiologist was 0.72<br>• Kappa ranged from a minimum of 0.57 to a maximum of 0.90.<br>• Overall, sensitivity was 0.92, and specificity was 0.80

Sims, 2014(17)	***Spectrum of pediatric cardiac disease in Malawi, Africa***	Conference abstract: *Global Heart*	Descriptive study	Characterizes the variety of cardiac disease presenting in a paediatric cardiac clinic and hospital in Lilongwe, Malawi, from September 2011 to June 2012.	• Children with cardiac diagnoses (n = 210)<br>• 115 had congenital defects<br>• 95 had acquired defects.<br>• Of the children with RHD<br>• The majority had severe disease (62%)<br>• 43 children with RHD had mitral regurgitation, 32 had mitral stenosis, 19 had aortic regurgitation, 12 children had aortic stenosis.<br>• Many children had more than one lesion.
Author, year	Study title	Literature type	Study design	Area evaluated	Major/relevant findings

Milhoan, Folsom & Kypuros, 2014(23)	***Challenges in determining prevalence of rheumatic heart disease: Lessons learned from scanning in remote areas***	Conference abstract: ***Annals of Pediatric Cardiology***	Cross-sectional	Mass screenings in schools and hospital/clinic settings with referral to a paediatric cardiologist if any positive signs of cardiac disease were revealed. The screening exam involved a history, physical exam, ECG, pulse oximetry and portable echocardiogram. The aim was to obtain a true prevalence of RHD in rural areas. Countries included were Mongolia, Iraq, Kosovo, Papua New Guinea, Mexico, Liberia and Malawi.	• Children were screened across 8 countries (n = 11, 190)<br>• In total, 27 cases of RHD were identified<br>• No part of the screening exam was found to be more sensitive than physical exam by stethoscope for diagnosing significant cardiac disease.<br>• Preliminary data using hand-held echocardiography increased the diagnoses of insignificant incidental cardiac findings e.g., trivial mitral regurgitation with a normal mitral valve.<br>• Utilizing handheld echocardiography has increased the diagnosis of RHD and may have led to improperly including physiologic mitral regurgitation with or

					without mitral valve prolapse within the reported prevalence.
Selman, Kennedy & Borgstein, 2013(18)	***Defining the burden of paediatric cardiac disease in Malawi-the experience from a tertiary referral centre***	Conference abstract: ***Archives of Disease in Childhood: Education and Practice Edition***	Descriptive study	Between January 2009 and February 2011, the age and cardiac diagnosis children with an abnormal echocardiogram attending a tertiary referral hospital in Malawi was recorded in a database. The range of diagnoses were described.	• Children (n = 250)<br>- 111 (44.4%) had acquired heart disease<br>• 22.4% RHD, 13.6% dilated cardiomyopathy<br>- 139 (55.6%) had congenital heart disease<br>• The mean age of presentation was 11 years 6 months for RHD.<br>• For RHD, most present late.<br>• The clinic provides monthly BPG injections for secondary prevention.<br>• 44 children underwent cardiac surgery abroad in specialist centres following referral from the clinic.

Zühlke et al., 2016(25)	***Clinical Outcomes in 3343 Children and Adults With Rheumatic Heart Disease From 14 Low- and Middle-Income Countries***	Journal article: *Circulation*	Cohort study	Two-year (January 2010-November 2012) follow-up study of 3343 patients from 25 centres across 14 low-to-middle-income countries to evaluate mortality and morbidity associated with RHD.	• RHD is associated with significant morbidity and mortality despite affecting a young patient cohort.<br>• Mortality was substantially higher in low-income countries at follow-up<br>• Education beyond primary school was associated with a 33% lower risk of death<br>• In the low-income countries (n=964)<br>- 200 (20.8%) died<br>- 87 (9%) had congestive heart failure<br>- 4 (0.4%) had a recurrence of ARF<br>- 14 (1.5%) had a stroke or TIA<br>- 1 (0.1%) had infective endocarditis<br>- 28 (2.9%) had atrial fibrillation<br>- 30 (3.1%) had had surgery

					• Loss to follow-up was 11.5% across all sites. Those lost were predominantly individuals with severe disease or with poorer education status.
Zühlke et. al, 2014(19)	***Characteristics, complications, and gaps in evidence-based interventions in rheumatic heart disease: the Global Rheumatic Heart Disease Registry (the REMEDY study)***	Journal article: *European Heart Journal*	Descriptive study	Documentation of patterns of RHD in terms of patient characteristics, treatments and disease outcomes in low-, lower-middle-, and upper-middle-income countries (January 2010- November 2012). Malawi was included as a low-income country.	• Participants (n = 3343) across 14 countries with symptomatic RHD were included<br>• 1110 participants were from low-income countries, which included Malawi.<br>• RHD patients in low-income countries are mostly young (median age: 24 (15-34) and female [728 (65.8%)]<br>• 630 (86.5%) of the women were of child-bearing age<br>• 405 (36.6%) were children<br>• 247 (22.3%) had a history of ARF

					• Patients with RHD from low-income countries have a high unemployment rates [529 (75.4%)]
Soliman & Juma, 2008(28)	***Cardiac disease patterns in northern Malawi: epidemiologic transition perspective***	Journal article: *Journal of epidemiology*	Retrospective analysis	Documented cardiovascular disease (CVD) patterns in Northern Malawi (January 2001-August 2005).	• Patients (n = 3908) aged 2 months - 82 years attending the outpatient cardiology clinic of Mzuzu Central Hospital were included in the 5-year register period:<br>• The most common presentation of CVD in this tertiary centre was RHD (n = 1176, 30%)<br>• Other common patterns were hypertensive heart disease and cardiomyopathy
Author, year	Study title	Literature type	Study design	Area evaluated	Major/relevant findings
Allain et. al, 2016(9)	***The Spectrum of Heart Disease in adults in***	Journal Article:	Literature review	Reviewing the available literature from a number of countries in Sub-	• Echocardiography is a versatile and cost-effective clinical tool that can be

	Malawi: A review of the literature with reference to the importance of echocardiography as a diagnostic modality	*Malawi Medical Journal*		Saharan Africa to highlight the utility of echocardiography as a diagnostic and research tool in resource-poor settings.	used to diagnose and monitor cardiac manifestations of both communicable and non-communicable diseases in Malawi.
Republic of Malawi Ministry of Health: The Malawi Noncommunicable Diseases & Injuries Poverty Commission(37)	***The Malawi Noncommunicable Diseases & Injuries Poverty Commission Report***	Government report		Assessing burden of NCDIs, including RHD, in Malawi and the cost-effectiveness of interventions	• The National NCDI Poverty Commission was set up in 2016 to assess the burden of NCDIs in Malawi, to prioritise strategies that address this burden and to identify resource gaps with a focus on the poorest portions of the population
NCD and Mental Health Unit, Ministry of Health, Malawi(38)	***National Action Plan for the Prevention and Management of Non-Communicable Diseases in Malawi (2017-2022)***	Government report		Defines RHD among a list of priority conditions in Malawi	• The report outlines broadly some strategies for reducing the burden of various NCDs in Malawi. While RHD is mentioned as a priority, there are no

					suggestions made as to appropriate interventions that are specific to RHD.
ClinicalTrials.gov (ID: NCT02832544)(40)	***Investigation of Rheumatic AF Treatment Using Vitamin K Antagonists, Rivaroxaban or Aspirin Studies, Non-Inferiority***	Clinical trial study record	Prospective randomised, parallel group, open-label clinical trial	International, multicentre trial to evaluate if rivaroxaban is non-inferior (or superior) to VKA in patients with RHD, atrial fibrillation/flutter and stroke risk.	• Includes 4565 patients from 138 sites, including three central hospitals in Malawi<br>• Study completed in 2022 – VKA led to lower rates of cardiovascular events or death than rivaroxaban in RHD patients with concomitant atrial fibrillation. The rate of bleeding was also not higher in warfarin groups compared to rivaroxaban groups.
Karthikeyan et. al, 2024(29)	***Mortality and Morbidity in Adults With Rheumatic Heart Disease***	Journal article: *JAMA*	Prospective observational study	Multi-centre, hospital-based study assessing the risk and predictors of mortality and morbidity in clinically significant RHD across a number of	• Includes 13,696 patients enrolled between August 2016 and May 2022 from 138 sites in 24 RHD-endemic countries

				lower-to-middle-income countries including Malawi.	• Mortality from clinical RHD was the highest in LICs<br>- 7% per year (compared to 5% overall mortality)<br>- HF and sudden cardiac death accounted for the majority of RHD-associated deaths across income groups<br>• RHD mortality is increased with more severe valvular disease, the strongest predictors of which were CHF and pulmonary arterial hypertension at baseline<br>• 30-day mortality for participants hospitalised with HF was higher in

					LICs than in middle-income countries (MICs)<br>• Complications of RHD in LICs relative to MICs<br>- Moderate to severe mitral/aortic regurgitation was more common in LICs<br>- Severe aortic stenosis and atrial fibrillation were more common in MICs<br>- Complications of RHD including stroke, infective endocarditis and recurrences of RF are rare in LICs<br>• RHD mortality was reduced in participants with a history of valvuloplasty and valve surgery

					- Attainment of valvuloplasty or valve surgery was lower in LICs (3.3%) than lower-middle- (4%) and upper-middle-income countries (7.2%)<br>• RHD mortality was reduced among patients using secondary antibiotic prophylaxis<br>- The use of secondary antibiotics was higher in LICs than MICs
