# Supplementary figures and images for: The burden of rheumatic heart disease and issues affecting the provision of care in Malawi: A scoping review
Source: PLoS Negl Trop Dis. 2025 Aug 19;19(8):e0013400. doi: 10.1371/journal.pntd.0013400 (PMC12380299; doi:10.1371/journal.pntd.0013400)

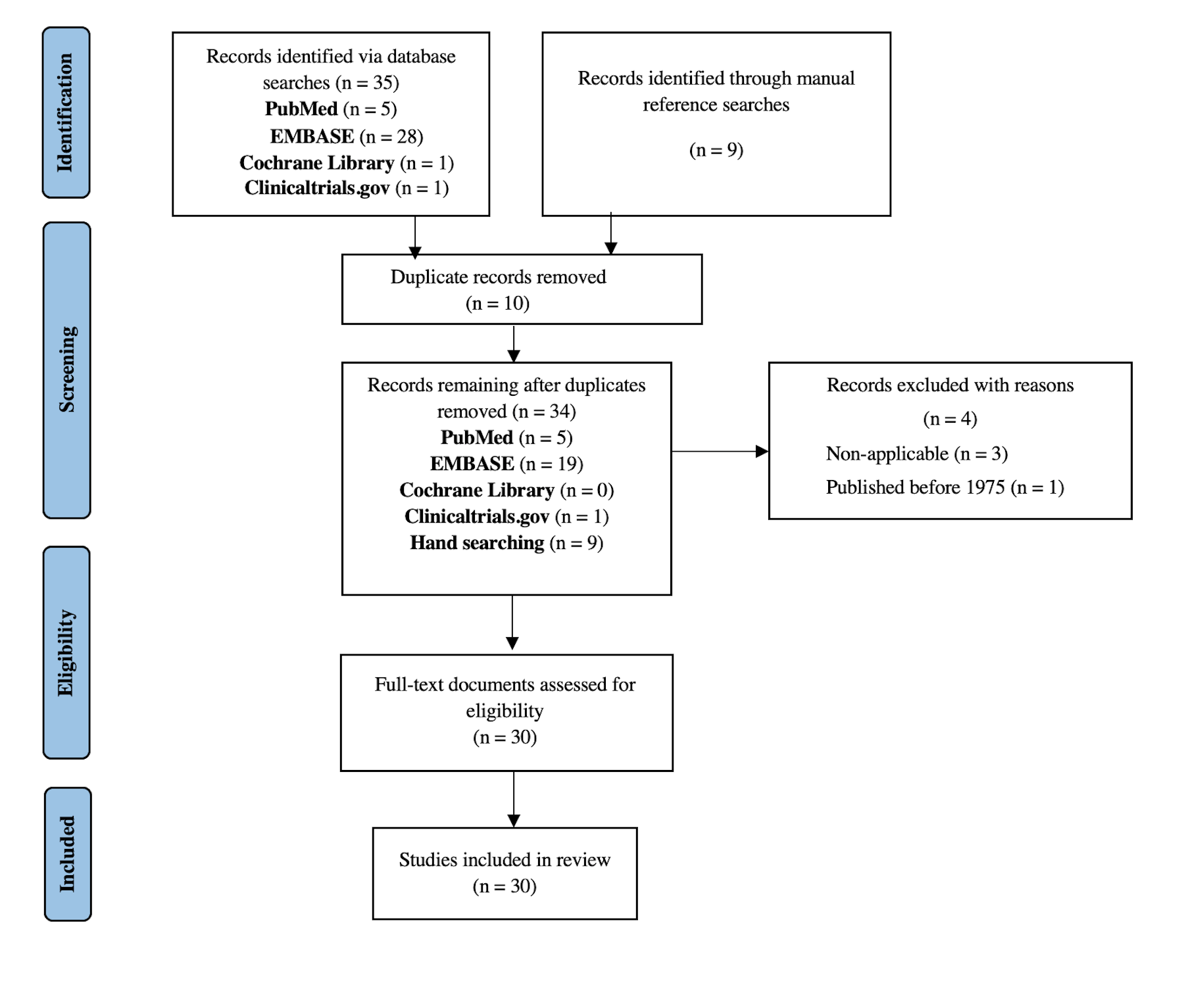

Supplement: S1 Fig — Flow diagram illustrating the identification, screening, eligibility assessment, and inclusion of sources in accordance with the PRISMA-ScR (Preferred Reporting Items for Systematic reviews and Meta-Analyses extension for Scoping Reviews) guidelines. (TIF) [file pntd.0013400.s004.tif]

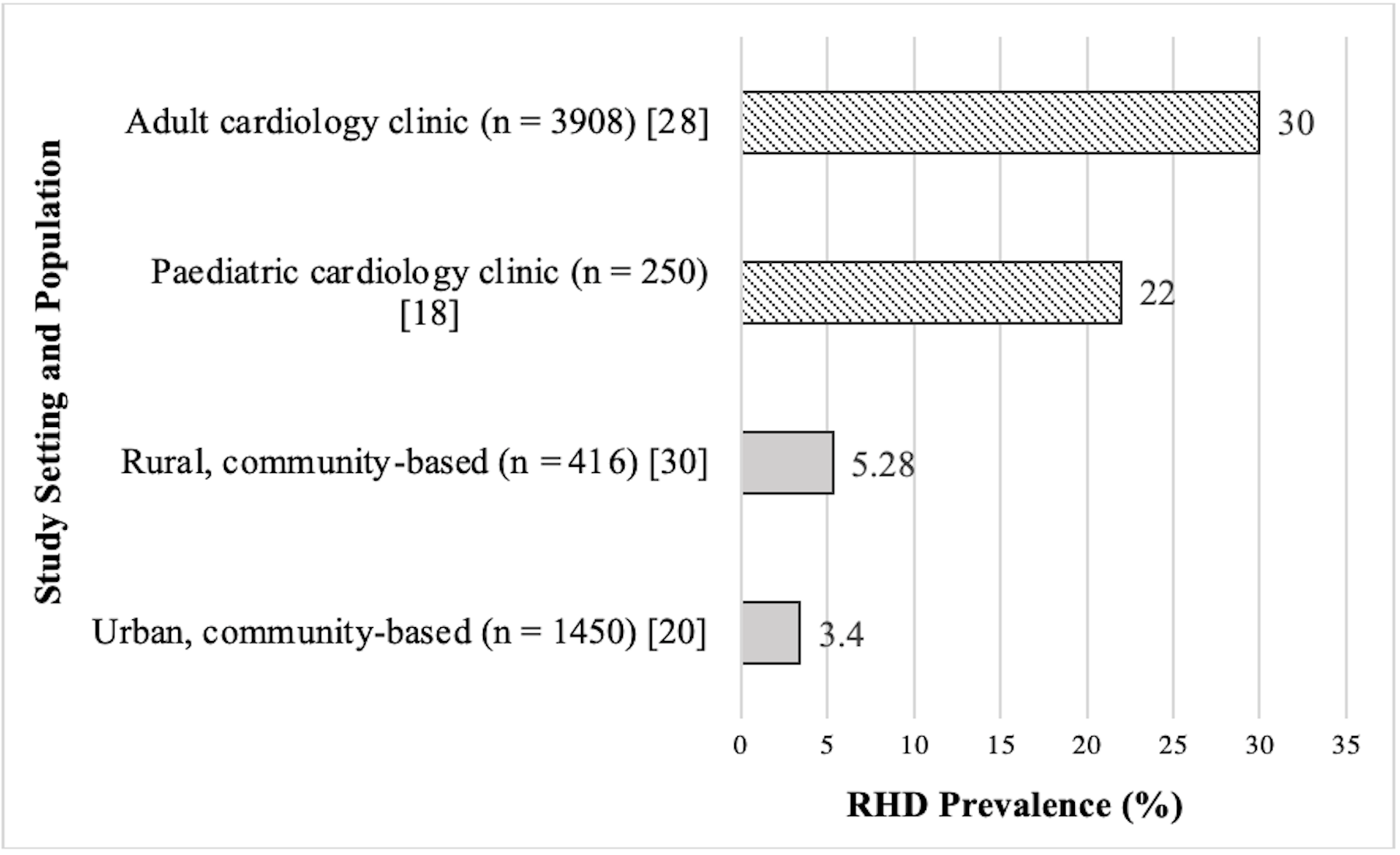

Supplement: S2 Fig — Prevalence of rheumatic heart disease (RHD) (%) reported across four study settings: adult cardiology clinic, paediatric cardiology clinic, rural community-based, and urban community-based populations. Sample sizes (n) and reference numbers correspond to the included studies. (TIF) [file pntd.0013400.s005.tif]
